# Cardiotoxicity in Cancer Patients: The Prevalence, Risk Factors, and Cardioprotective Measures in a Cancer Centre in Saudi Arabia

**DOI:** 10.7759/cureus.59608

**Published:** 2024-05-03

**Authors:** Ahmed M Badheeb, Yahya Alhosni, Mohammed Alshahrani, Tariq A Asery, Saeed M Al nasher, Islam A Seada, Abdullah M Nasher, Bandar A Alotaibi, Esraa Alsaaed, Alhassan M Alsalem, Abdullah Abu bakar, Waleed Alselwi, Faisal Ahmed, Nasher H Alyami, Lotfi Bin Dahman, Mohamed Badheeb, Hamoud Y Obied

**Affiliations:** 1 Medicine, Hadhramout University, Mukalla, YEM; 2 Oncology, King Khalid Hospital, Najran, SAU; 3 Cardiology, King Khalid Hospital, Najran, SAU; 4 Internal Medicine, King Khalid Hospital, Najran, SAU; 5 Internal Medicine, King Khalid Hospital, Riyadh, SAU; 6 Internal Medicine, King Khalid Hospital, Abha, SAU; 7 Cardiothoracic Surgery, King Khalid Hospital, Najran, SAU; 8 Ophthalmology, King Khalid Hospital, Najran, SAU; 9 Medical Oncology, King Fahad Specialist Hospital, Dammam, SAU; 10 Urology, Ibb University, Ibb, YEM; 11 Laboratory Medicine, Hematology Unit, Najran General Hospital, Ministry of Health, Najran, SAU; 12 Clinical Biochemistry, Hadhramout University, Mukalla, YEM; 13 Internal Medicine, Yale New Haven Health, Bridgeport Hospital, Bridgeport, USA; 14 Surgery, Faculty of Medicine, Najran University, Najran, SAU; 15 Cardiac Surgery, King Khalid Hospital, Najran, SAU

**Keywords:** najran, saudi arabia, protective factor, predictive factors, cancer, anticancer, cardiotoxicity

## Abstract

Background: Chemotherapy-related cardiotoxicity can exhibit several patterns of functional, structural, and vascular complications. This study aims to identify the patterns and the factors associated with cardiotoxicity in cancer patients.

Method: A retrospective cross-sectional analysis of 96 adult cancer patients undergoing anticancer therapy was investigated at King Khalid Hospital in Najran, Saudi Arabia, from May 2022 to April 2023. The data on patient and cancer characteristics, treatment, and outcomes were collected and analyzed. Factors associated with cardiotoxicity were investigated through univariate analyses using odds ratio (OR) and 95% confidence interval (CI).

Results: Among the 96 cancer patients in the study, cardiotoxicity occurred in 12 individuals (12.5%). The mean age was 57.0 ± 13.3 years (range: 32-81 years), with 32 (33.3%) being above 65 years. The most common comorbidities were diabetes (n=48; 50%), followed by hypertension (n=32; 33.3%), and dyslipidemia (n=20; 20.8%). The most common cancers were gastrointestinal cancer (n=32; 33.3%), followed by breast cancer (n=22; 22.9%) and lymphoma (n=14; 14.6%). Females were disproportionately affected (64.6%), with 57.3% of them in the metastatic stage. The majority of patients (90.6%) had normal ejection fraction before chemotherapy initiation. In univariate analysis, current smoking (OR: 7.00; 95%CI: 1.94-25.25, p= 0.003), history of percutaneous cardiac intervention (OR: 40.24; 95%CI: 1.80-896.26, p= 0.019), diabetes (OR: 6.05; 95%CI: 1.24-29.32, p= 0.025), renal failure (OR: 8.20; 95%CI: 0.91-74.88, p= 0.046), dyslipidemia (OR: 5.00; 95 CI: 1.38-18.32, p=0.012), anthracycline use (OR: 18.33; 95%CI: 4.36-126.55, p <0.001), trastuzumab use (OR: 25.00; 95%CI: 6.25-129.86, p < 0.001), and increased chemotherapy cycles number (> 10 cycles) (OR: 73.00; 95%CI: 8.56- 622.36, p < 0.001) were associated with cardiotoxicity. Additionally, beta-blocker use was associated with lower rates of cardiotoxicity (OR: 0.17; 95%CI: 0.036-0.84, p= 0.029).

Conclusions: The incidence of cardiotoxicity among cancer patients treated with chemotherapy is modest, difficult to predict, and independent of baseline cardiac systolic functions. Factors associated with cardiotoxicity include smoking, history of percutaneous cardiac intervention, diabetes, renal failure, dyslipidemia, anthracycline or trastuzumab use, and increased chemotherapy cycle numbers. A combination of various anticancer drugs and chemotherapy may dramatically raise the risk of cardiotoxicity in cancer patients. As a result, patients receiving high-risk cardiotoxic drugs should be monitored with caution to avoid drug-related cardiotoxicity. Furthermore, proactive treatment techniques aiming at reducing the possible cardiotoxic effects of anticancer therapy are critical.

## Introduction

Chemotherapy-related cardiotoxicity gained significant emphasis as it has been linked to the risk of cardiac dysfunction, thromboembolic events, and arrhythmias, necessitating the implementation of effective prevention and treatment strategies [1]. Notably, cardiotoxicity has become more noticeable with the advancement of cancer screening and detection, along with the improvement of chemotherapeutic efficacy and overall prolonged survival [2]. Several pathogenic mechanisms have been proposed by which chemotherapeutic agents can result in cardiac dysfunction, including maladaptive pro-inflammatory effects with subsequent remolding. Additionally, the formation of reactive oxygen species has been linked to mitochondrial dysfunction and permanent cardiomyocyte damage. While prior reports have shown significant association with anthracyclines and taxanes, the wider use of targeted therapy has shed light on the cardiac alteration that has been observed with several immunological agents (e.g., trastuzumab and sunitinib) [3].

A recent survey in Najran, Saudi Arabia, on the prevalence of cardiotoxicity among 78 patients receiving anticancer therapy reported alarming figures of 12% of cardiotoxicity occurrence [4]. Additionally, dyslipidemia, previous heart, and impaired baseline ejection fraction (EF) were associated with an increased risk of cardiotoxicity. Furthermore, statin and antiplatelet agents were protective agents against cardiotoxicity. Previous research has found a conflicting relationship between anticancer treatment and drug-related cardiotoxicity, whether in single or combination regimens [4-6]. Most studies used relative risks (RRs) or odds ratios (ORs) to assess anticancer-induced cardiotoxicity. However, these investigations failed to account for the interval between the start of anticancer therapy and the onset of cardiotoxicity during the treatment period, which may have influenced the consistency of their results [4,6]. Additionally, the incidence of cardiotoxicity and adherence to monitoring recommendations remains poorly understood in Saudi Arabia. Thus, this study aims to investigate the prevalence of cardiotoxicity and factors associated with cardiotoxicity occurrence in adult cancer patients receiving anticancer therapy at the King Khalid Hospital in Najran, Saudi Arabia.

## Materials and methods

This was a retrospective cross-sectional study that included 96 adult cancer patients aged ≥18 who were treated with anticancer therapy (chemotherapy) at the oncology center of King Khalid Hospital in Najran, Saudi Arabia, between May 2022 to April 2023. The patients were assessed for left ventricular ejection fraction (LVEF) before, during, and after initiation of anticancer therapy. Patients who experienced cardiotoxicity during treatment were allowed to continue their remaining treatment, either by removing the medication from their regimen or by transitioning into an alternative regimen. Patients who did not undergo LVEF testing before or after administering anticancer therapy, and elderly patients with advanced or terminal diseases were excluded.

Study protocol and main outcome

The study evaluated the patients on their baseline for their sociodemographic, comorbidities, site, and stage of cancer. In addition, LVEF was reviewed prior to initiating anti-cancer chemotherapy and on the follow-up duration (three to six months). The presence of cardiotoxicity was determined using the criteria set forth by Guglin et al., which included an LVEF measurement that fell below 50% or a decrease in LVEF of 10% or more from the initial measurement [7]. Additionally, symptomatic heart failure was considered a factor, even in cases where there was no decline in LVEF. The principal outcome encompassed the prevalence of cardiotoxicity throughout anticancer therapy. The secondary outcome aimed to ascertain the prognostic factors and protective agent determinants for cardiotoxicity.

Sample size

The sample size was determined using the single population proportion method under the following assumptions: the proportion of cardiotoxicity was obtained from research done by Hamirani et al. (12.5%, n= 27/216), with a 95% confidence interval (CI), margin error of 5, and 5% non-response rate [5]. Finally, the current study's sample size of 96 was determined utilizing the systematic sampling approach.

Data collection

Data from electronic records and medical charts of eligible cancer patients were collected using a structured format. The collected information included patient demographics (age, gender, and current smoking history), comorbidities (diabetes mellitus, heart disease, hypertension, previous percutaneous cardiac interventions, hyperlipidemia, trastuzumab use, anthracycline use, and renal failure), cancer type, stage (metastatic versus nonmetastatic), drug regimen, chemotherapy cycle number, use of cardiac protective agents, and pre-and post-treatment LVEF values. The anticancer therapy agent was grouped into monoclonal agents, alkylating agents, anthracyclines agents, and taxans. The cardiac protection agents were grouped into beta-blockers, angiotensin-converting enzyme (ACE) inhibitors, statins, and antiplatelets [4].

Statistical analysis

Categorical data were reported as frequency (percentage), and continuous variables as mean ± standard deviation (SD). All parameters were considered significant if their 95%CIs did not contain zero. To find independent risk variables for cardiotoxicity, the T-test or Mann-Whitney test was used for numerical variables, and Chi-square or Fisher's exact test for categorical variables. ORs and their corresponding 95%CIs were calculated from the b coefficients and standard errors. A significance level of p < 0.05 was considered statistically significant. The statistical analysis was performed using IBM SPSS Statistics for Windows, Version 20.0 (Released 2011; IBM Corp., Armonk, New York, United States).

Ethical approval

The study was approved by the Ethics Research Committees of King Khaled Hospital (Code: KACST, KSA: H-I1-N-086), in compliance with the ethical standards outlined in the Declaration of Helsinki. Due to the anonymous retrospective nature of the study, written informed consent from the included patients was not required.

## Results

Among the 96 cancer patients included in the study, cardiotoxicity occurred in 12 individuals (12.5%). The mean age was 57.0 ± 13.3 years (range: 32-81 years), with 32 (33.3%) being above 65 years. The most common comorbidities were diabetes (n=48; 50.0%), followed by hypertension (n=32; 33.3%), and dyslipidemia (n=20; 20.8%). The most common cancers were gastrointestinal cancer (n=32; 33.3%), followed by breast cancer (n=22; 22.9%) and lymphoma (n=14; 14.6%), respectively. Most cases (n=62; 64.6%) were female and in the metastatic stage (n=55; 57.3%). Eighty-seven cases (90.6%) had a normal ejection fraction before starting therapy. Characteristics of study participants are mentioned in Table 1.

**Table 1:** Characteristics of study participants

Characteristic	Subgroup	Frequency (Percentage)
Age (year)	Mean ±SD	57.0 ±13.3
	≤ 65 years	64 (66.7%)
	> 65 years	32 (33.3%)
Gender	Male	34 (35.4%)
	Female	62 (64.6%)
Smoking	-	21 (21.9%)
Comorbidities	Hypertension	
	Diabetes	48 (50.0%)
	Heart disease	6 (6.2%)
	Renal failure	4 (4.2%)
	Dyslipidaemia	20 (20.8%)
	Percutaneous cardiac intervention	2 (2.1%)
Site of cancer	Gastrointestinal cancers	32 (33.3%)
	Breast cancers	22 (22.9%)
	Lymphoma	14 (14.6%)
	Urologic cancers	13 (13.5%)
	Gynecological cancers	8 (8.3%)
	Other cancers	7 (7.3%)
Cancer stage	Metastatic	55 (57.3%)
	Nonmetastatic	41 (42.7%)
Baseline ejection fraction	≥ 55%: Normal	87 (90.6%)
	50% to 54%: Borderline low	1 (1.0%)
	≤ 49%: Impaired	8 (8.3%)
Cardiotoxicity	Yes	12 (12.5%)
	No	84 (87.5%)

Regarding treatment used, monoclonal anticancer was the most administrated medication in 47 (49.0%) cases, followed by alkylating agent in 38 (39.6%) cases, anthracycline agents in 28 (29.2%) cases, and trastuzumab in 18 (18.8%) cases. Most cases (n=46; 47.9%) received less than five cycles. The most cardiac protective agents were beta blockers, ACE inhibitors, and statins in 47 (49.0%), 35 (36.5%), and 33 (34.4%), respectively (Table 2).

**Table 2:** Chemotherapy and cardiac protective agents' characteristics of study participants RCVP: rituximab with cyclophosphamide, vincristine, and prednisone; RCHOP: rituximab with cyclophosphamide, doxorubicin, vincristine, and prednisone, AC: doxorubicin and cyclophosphamide; FOLFOX: leucovorin, 5-fluorouracil, and oxaliplatin

Variables	Frequency (Percentage)
Chemotherapy group	
Monoclonal anticancer use	47 (49.0%)
Alkylating agent users	38 (39.6%)
Anthracycline agent users	28 (29.2%)
Taxane users	12 (12.5%)
Trastuzumab users	18 (18.8%)
Protective cardiotoxicity agents	
Betablocker	47 (49.0%)
ACE inhibitors	35 (36.5%)
Statins	33 (34.4%)
Antiplatelets	16 (16.7%)
Number of chemotherapy cycles	
≤ 5	46 (47.9%)
6-10	28 (29.2%)
> 10	22 (22.9%)
Chemotherapy protocols	
R-CVP	3 (3.1%)
Docetaxel	6 (6.2%)
FOLFOX	15 (16%)
RCHOP	2 (2.1%)
AC	12 (12%)
Carbo taxol	3 (3.1%)
XELOX	6 (6.2%)
Paclitaxel	4 (4.2%)
Other	45 (47%)

Factors associated with cardiotoxicity

In univariate analysis, current smoking (OR: 7.00; 95%CI: 1.94-25.25, p= 0.003), history of percutaneous cardiac intervention (OR: 40.24; 95%CI: 1.80-896.26, P= 0.019), diabetes (OR: 6.05; 95%CI: 1.24-29.32, p= 0.025), renal failure (OR: 8.20; 95%CI: 0.91-74.88, p=0.046), dyslipidemia (OR: 5.00; 95%CI: 1.38-18.32, p=0.012), Anthracycline (OR: 18.33; 95%CI: 4.36-126.55, p <0.001), trastuzumab use (OR: 25.00; 95%CI: 6.25-129.86, p < 0.001), and increased chemotherapy cycles number (> 10 cycles) (OR: 73.00; 95%CI: 8.56-622.36, p < 0.001) were associated with cardiotoxicity (Table 3).

**Table 3:** Univariate analysis of factors associated with cardiotoxicity

Characteristic	Subgroup	Cardiotoxicity (N=12)	No cardiotoxicity (N=84)	OR (95 % CI)	p-value
Age (year)	≤ 65	5 (41.7)	59 (70.2)	Reference group	0.058
	> 65	7 (58.3)	25 (29.8)	3.30 (0.95-11.41)	
Gender	Male	4 (33.3)	30 (35.7)	Reference group	0.871
	Female	8 (66.7)	54 (64.3)	1.11(0.32-3.99)	
Smoking	No	5 (41.7)	70 (83.3)	Reference group	0.003
	Yes	7 (58.3)	14 (16.7)	7.00 (1.94-25.25)	
Hypertension	No	6 (50.0)	58 (69.0)	Reference group	0.198
	Yes	6 (50.0)	26 (31.0)	2.23 (0.66-7.57)	
History of percutaneous cardiac intervention	No	10 (83.3)	84 (100.0)	Reference group	0.019
	Yes	2 (16.7)	0 (0.0)	40.24 (1.80-896.26)	
Diabetes	No	2 (16.7)	46 (54.8)	Reference group	0.025
	Yes	10 (83.3)	38 (45.2)	6.05 (1.24-29.3218)	
Chronic heart disease	No	10 (83.3)	80 (95.2)	Reference group	0.135
	Yes	2 (16.7)	4 (4.8)	4.00 (0.51-23.45)	
Renal failure	No	10 (83.3)	82 (97.6)	Reference group	0.046
	Yes	2 (16.7)	2 (2.4)	8.20 (0.91-74.88)	
Dyslipidaemia	No	6 (50.0)	70 (83.3)	Reference group	0.012
	Yes	6 (50.0)	14 (16.7)	5.00 (1.38-18.32)	
Cancer stage	Non-metastatic	10 (83.3)	31 (36.9)	Reference group	0.07
	Metastatic	2 (16.7)	53 (63.1)	0.12 (0.02-0.48)	
Baseline ejection fraction	≥ 45%	10 (83.3)	78 (92.9)	Reference group	0.279
	< 45%	2 (16.7)	6 (7.1)	2.60 (0.4607-14.6724)	
Monoclonal anticancer use	No	5 (41.7)	44 (52.4)	Reference group	0.487
	Yes	7 (58.3)	40 (47.6)	1.54 (0.46-5.57)	
Alkylating agent users	No	6 (50.0)	52 (61.9)	Reference group	0.430
	Yes	6 (50.0)	32 (38.1)	1.62 (0.47-5.62)	
Anthracyclines users	No	2 (16.7)	66 (78.6)	Reference group	<0.001
	Yes	10 (83.3)	18 (21.4)	18.33 (4.36-126.55)	
Hormonal agent use	No	12 (100.0)	82 (97.6)	Reference group	0.860
	Yes	0 (0.0)	2 (2.4)	1.32 (0.059-29.13)	
Taxan users	No	11 (91.7)	73 (86.9)	Reference group	0.644
	Yes	1 (8.3)	11 (13.1)	0.60 (0.03 to 3.60)	

Protective factors against cardiotoxicity

In univariate analysis, only beta-blocker use was a protective factor against cardiotoxicity and was statistically significant (OR: 0.17, 95%CI: 0.036-0.84, p= 0.029) (Table 4).

**Table 4:** Univariate analysis of protective factors against cardiotoxicity ACE: angiotensin-converting enzyme

Characteristic	Subgroups	Cardiotoxicity (N=12)	No cardiotoxicity (N=84)	OR (95 % CI)	p-value
Antiplatelets	No	9 (75.0)	71 (84.5)	Reference group	0.4129
	Yes	3 (25.0)	13 (15.5)	0.55 (0.13-2.31)	
Betablocker	No	10 (83.3)	39 (46.4)	Reference group	0.029
	Yes	2 (16.7)	45 (53.6)	0.17 (0.036-0.84)	
ACE inhibitors	No	10 (83.3)	51 (60.7)	Reference group	0.145
	Yes	2 (16.7)	33 (39.3)	0.31 (0.063-1.500)	
Statins	No	10 (83.3)	53 (63.1)	Reference group	0.183
	Yes	2 (16.7)	31 (36.9)	0.34 (0.070-1.662)	

## Discussion

In this study, we investigated the prevalence and factors influencing cardiotoxicity among cancer patients treated with anticancer therapy. Our findings revealed that smoking, history of percutaneous cardiac intervention, diabetes, renal failure, dyslipidemia, anthracycline, and trastuzumab use, and increased chemotherapy cycle numbers (> 10 cycles) were all associated with cardiotoxicity.

In this study, the prevalence of cardiotoxicity was 12.5%. The result was similar to our previous report of 12% cardiotoxicity occurrence among cancer patients during anticancer treatment in Najran City [4]. Prior studies have shown variable prevalences of cardiotoxicity. In a recent systemic review, the prevalence of cardiotoxicity was 8.3% among cancer patients who received chemotherapy [8]. Comparatively, Curigliano et al. reported lower rates of cardiotoxicity in individuals treated with trastuzumab, reaching 3.8%, while cardiotoxicity with cyclophosphamide ranged from 7% to 28% [9]. Furthermore, Cho et al. reported a cumulative incidence of cardiotoxicity of 6.1% at two years, with no significant change from about nine months, and 20.2% at two years, with no significant change from about 15 months, after starting doxorubicin-containing therapy without and with trastuzumab, respectively [10]. The cardiotoxicity prevalence can be influenced by chemotherapy protocol, duration, and multiple anticancer drug use. In general, cardiotoxicity incidence among cancer patients varies significantly, with rates ranging from 3.8% to 37.5% [9,11]. The slightly higher rate of reported cardiotoxicity rates in our study might be attributed, in part, to the smaller sample size, along with patient factors such as advanced tumor stage, comorbidities, differences in chemotherapy regimen, and types of cancer that may raise the risk of cardiotoxicity.

A comprehensive safeguarding plan for cardiotoxicity prevention is critical, balancing the risks and benefits of chemotherapy, as well as regular monitoring of heart function and biomarkers.

In this study, cardiotoxicities were increased with increasing age. However, older age was not an independent factor for cardiotoxicities. This might be explained by the higher prevalence of several comorbidities that make the patients prone to the cardiotoxic effects of cancer therapy [12]. Additionally, cardiotoxicities were more prevalent in the female gender. However, the difference was not statistically significant. The findings may be due to the low sample size in our study. A similar result of less importance on age and gender was mentioned by Kobat et al. [13]. Other studies have found advanced age [14] and female sex [15] as risk factors for cardiotoxicities.

In this study, diabetes was a risk factor for cardiotoxicity in cancer patients. Similarly, other studies found that diabetic cancer patients were at a greater risk compared to non-diabetic patients [4,13]. Given that diabetic individuals already have elevated inflammation-associated protein expression in the heart, increasing oxidative stress can combine with anthracyclines to aggravate cardiac damage [16]. Nevertheless, there is no strong evidence to substantiate such a correlation.

Patients with pre-existing cardiac conditions are more likely to develop cardiotoxicity or other cardiac events that may necessitate treatment changes, dose adjustments, or even early treatment discontinuation [17,18]. Similarly, in this study, cardiotoxicity was more commonly observed among patients with a prior history of percutaneous cardiac interventions and was statistically significant. Another study reported a higher prevalence of cardiovascular events among individuals with a history of heart disease and impaired baseline EF [4]. However, in this study, a history of chronic heart failure, impaired bassline EF, and hypertension were not associated with cardiotoxicity. These findings may be limited due to the small sample size. Additionally, the study's scope was restricted to patients with impaired systolic function, without stratifying them based on medical optimization or adherence to guideline-directed medical therapy. Thus, the generalizability of these findings might be limited.

Dyslipidemia was shown to be a statistically significant risk factor for cardiotoxicity in this study. This is due to the oxidative stress generated by hyperlipidemia. Jia et al. found that palmitate treatment in H9C2 cardiomyocytes enhanced apoptosis due to oxidative stress generated by reactive oxygen species (ROS) produced during lipid peroxidation [19]. The findings of our study were comparable to prior research [16].

Our analysis suggests an increase in the risk of cardiotoxicity with smoking (the chance of cardiotoxicity was seven times higher in smokers than in nonsmokers), presumably because of the chemicals produced by smoke inhalation. A prior study found that smoking has a significant impact on anthracycline-induced cardiotoxicity as cigarette smoke elevated levels of two chemicals associated with cardiac shrinkage [20]. Similarly, a recent meta-analysis found that studies that focused just on OR corroborated a link between smoking and anthracycline-induced cardiotoxicity [16]. Additionally, smoking has been associated with higher chemotherapy toxicity and worse overall results [21]. 

In this study, the cardiotoxicity was higher among anthracycline and trastuzumab users. These findings are similar to prior reports [22]. Anthracyclin-induced cardiotoxicity is believed to be primarily related to excessive ROS production, leading to microsomal lipid peroxidation, ultrastructural changes, mitochondrial damage, and irreversible damage to cardiomyocytes [23]. In contrast, trastuzumab's cardiotoxicity represents a reversible and more favorable entity with no associated structural alterations

In this study, renal failure was associated with cardiotoxicity. According to Russo et al., the prevalence of trastuzumab-induced cardiotoxicity varies with the severity of renal disease (RD), ranging from 15% (in patients in class I RD) to 38% (in patients in class III RD). However, hypertension was not associated with cardiotoxicity [24].

The development of more effective anticancer therapy medications has surely improved cancer patient outcomes. However, it is critical to recognize that these medications might have serious short- and long-term side effects [25]. Individuals exposed to chemotherapeutic medicines known to enhance the risk of heart failure, such as anthracyclines, trastuzumab, sunitinib, and sorafenib, should have their heart functions assessed. This screening should adhere to the American College of Cardiology/American Heart Association standards [26,27]. In this study, we observed that those who completed more than 10 chemotherapy cycles were more susceptible to anti-cancer cardiotoxicity. These findings are consistent with earlier studies indicating that cumulative dosage was the strongest predictive risk factor [4,28].

There are studies exploring the use of beta-blockers, ACE inhibitors, or ARBs as preventative treatments for cancer patients to reduce cardiotoxicity [4,29]. In the current study, patients who received beta-blockers had a relatively lower risk for cardiotoxicity compared to non-receivers; this difference was statistically significant. Additionally, in this study, receiving anti-palate, ACE inhibitors, and statin reduced the cardiotoxicity but was not statistically significant.

Study Limitations

This retrospective investigation was carried out at a single center which restricts the findings' generalizability to other contexts. Furthermore, the low sample size was another limitation. As a result, the study's findings apply to a specific group of cancer survivors and their cardiovascular investigations (in some cases, the OR was high and the CI was very high. Therefore, the results should be interpreted with caution). Furthermore, it is vital to recognize that the criteria for determining cardiotoxicity may differ throughout healthcare facilities, emphasizing the lack of a generally standardized examination. This variability makes it difficult to correctly compare and understand data from different investigations. Furthermore, the study did not alter or categorize patients based on additional variables such as total cumulative dosage, infusion rate, speed, or cardiovascular risk factor management. These variables may impact the development of cardiotoxicity and should be examined in future studies. To increase understanding in this field, more large-scale research is needed to answer more specific issues and develop the best methods for screening cancer survivors.

## Conclusions

The incidence of cardiotoxicity among cancer patients treated with chemotherapy is modest, difficult to predict, and independent of baseline cardiac systolic functions. Factors associated with cardiotoxicity include smoking, history of percutaneous cardiac intervention, diabetes, renal failure, dyslipidemia, anthracycline or trastuzumab use, and increased chemotherapy cycle numbers. A combination of various anticancer drugs and chemotherapy may dramatically raise the risk of cardiotoxicity in cancer patients. As a result, patients receiving high-risk cardiotoxic drugs should be monitored with caution to avoid drug-related cardiotoxicity. Furthermore, proactive treatment techniques aiming at reducing the possible cardiotoxic effects of anticancer therapy are critical.
